# Metabolism and Pharmacokinetics of the Anti-Tuberculosis Drug Ethionamide in a Flavin-Containing Monooxygenase Null Mouse

**DOI:** 10.3390/ph5111147

**Published:** 2012-10-25

**Authors:** Amy L. Palmer, Virginia L. Leykam, Andrew Larkin, Sharon K. Krueger, Ian R. Phillips, Elizabeth A. Shephard, David E. Williams

**Affiliations:** 1 Department of Environmental and Molecular Toxicology, Oregon State University, 1007 ALS Corvallis, OR 97331, USA; Email: palmeram@onid.orst.edu (A.L.P.); virginia.leykam@oregonstate.edu (V.L.L.); larkinan@onid.orst.edu (A.L.); 2 Linus Pauling Institute, Oregon State University, 307 Linus Pauling Institute Corvallis, OR 97331, USA; Email: Sharon.krueger@oregonstate.edu; 3 School of Biological and Chemical Sciences, Queen Mary, University of London, London E1 4NS, UK; Email: i.r.phillips@qmul.ac.uk; 4 Department of Structural and Molecular Biology, University College London, London WC1E 6BT, UK; Email: e.shephard@ucl.ac.uk

**Keywords:** ethionamide, flavin-containing monooxygenase, *Mycobacterium tuberculosis*

## Abstract

Multiple drug resistance (MDR) in *Mycobacterium tuberculosis* (mTB), the causative agent for tuberculosis (TB), has led to increased use of second-line drugs, including ethionamide (ETA). ETA is a prodrug bioactivated by mycobacterial and mammalian flavin-containing monooxygenases (FMOs). FMO2 is the major isoform in the lungs of most mammals, including primates. In humans a polymorphism exists in the expression of FMO2. FMO2.2 (truncated, inactive) protein is produced by the common allele, while the ancestral allele, encoding active FMO2.1, has been documented only in individuals of African and Hispanic origin, at an incidence of up to 50% and 7%, respectively. We hypothesized that FMO2 variability in TB-infected individuals would yield differences in concentrations and ratios of ETA prodrug and metabolites. In this study we assessed the impact of the FMO2 genetic polymorphism on the pharmacokinetics of ETA after administration of a single oral dose of ETA (125 mg/kg) to wild type and triple *Fmo1/2/4*-null mice, measuring levels of prodrug *vs.* metabolites in plasma collected from 0 to 3.5 h post-gavage. All mice metabolized ETA to ETA *S*-oxide (ETASO) and 2-ethyl-4-amidopyridine (ETAA). Wild type mice had higher plasma concentrations of metabolites than of parent compound (*p* = 0.001). In contrast, *Fmo1/2/4*-null mice had higher plasma concentrations of parent compound than of metabolites (*p* = 0.0001). Thus, the human FMO2 genotype could impact the therapeutic efficacy and/or toxicity of ETA.

## 1. Introduction

Tuberculosis (TB), caused by *Mycobacterium tuberculosis* (mTB), has been recorded throughout history and continues to afflict the World populace. Approximately one-third of the World is infected [1]. In 2010, nearly nine million new infection cases and 1.4 million deaths related to TB occurred globally [1]. Complicating TB control is an increased incidence of multidrug resistant tuberculosis (MDR-TB) leading to increased use of second-line anti-TB drugs that can induce adverse drug reactions resulting in treatment noncompliance [2]. Ethionamide (ETA) is one of the more frequently prescribed second-line pharmaceuticals, and shares similarities in structure and antimicrobial function with isoniazid (INH), a first-line anti-TB drug [2,3,4].

The *M. tuberculosis* Rv3854c (*EtaA*) gene is a flavin monooxygenase homologous to human flavin-containing monooxygenases (FMOs, EC 1.14.13.8) [5]. Mutants of *EtaA* are known that display ETA resistance [6]. FMOs in mammals oxygenate ‘soft’ nucleophiles such as N-, S-, and P-containing drugs, pesticides, and dietary components [7,8,9,10] making them more excretable; this usually is a process of detoxification.

Of the five functional FMOs of humans, FMOs 1, 2, and 3 are most important in metabolism of xenobiotics. FMO1 has the broadest substrate specificity and, in humans, is located primarily in fetal liver, adult kidney, and intestine [10]. FMO3 is the primary FMO in adult human liver [10]. FMO2 is primarily located in the lung and expressed in most mammals [10], but humans have differing polymorphisms. The FMO2*2 allelic variant contains a C→T mutation resulting in a premature stop codon (CAG→TAG), and a truncated, inactive enzyme [10,11]. Occurrence of the FMO2*2 polymorphism differs dramatically between ethnic groups. All Europeans and Asians genotyped to date are homozygous for the FMO2*2 allele, while the ancestral allele, FMO2*1, encoding active full-length enzyme, is found in individuals of African and Hispanic origin in up to 50% and 7% of individuals, respectively [8,11,12,13,14]. The highest frequency of FMO2*1 is seen in sub-Saharan Africa, a region of the World that coincides with the highest TB incidence [15].

**Scheme 1:**
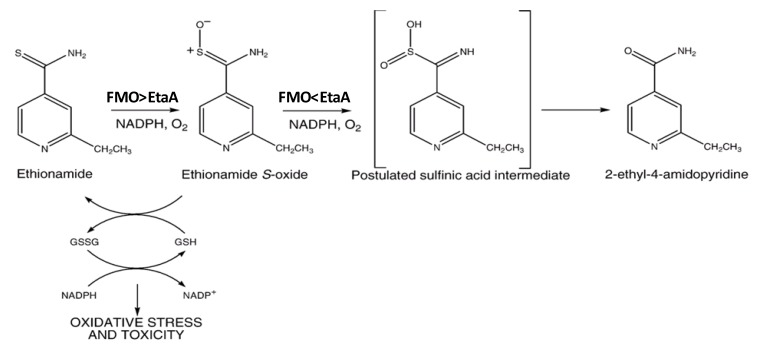
Proposed metabolism of ETA [16]. ETA is actively metabolized by human FMO2 and mycobacterial EtaA to its more reactive species of ETASO and the non-reactive 2-ethyl-4-amidopyridine decomposition product.

**Figure 1:**
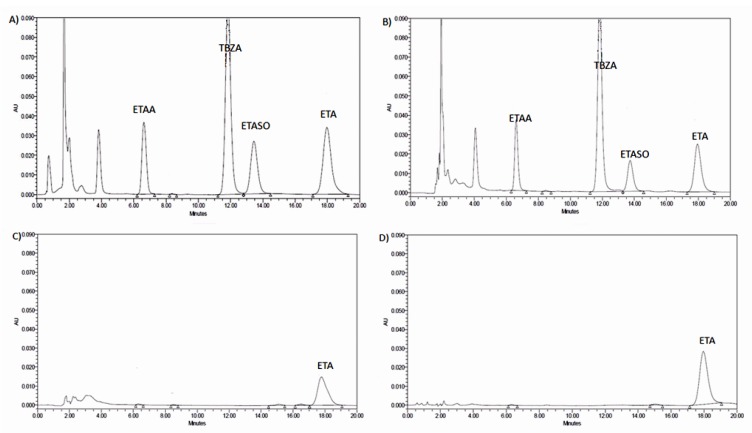
HPLC chromatogram tracings of freshly spiked and extracted samples from bovine plasma or vet syrup suspension. (**A**) Plasma spiked with 0.2 mM for each compound in the tracing shown. (**B**) Plasma spiked (0.15 mM, each) with ETA, its metabolites and TBZA is shown for a sample stored more than four months at −80 °C. (**C**) Vet syrup was spiked to make an ETA suspension (0.1 mM) that was extracted immediately after preparation. (**D**) Vet syrup spiked with an ETA suspension (0.1 mM) extracted following storage for more than one year at −80 °C. The absorbance for ETA, ETASO, ETAA, and TBZA were monitored at UV 267 nm.

Oxidative activation of the thioamide ETA by FMOs 1-3 occurs in mammals and mycobacteria [16,17,18,19,20] in two oxygenation steps that are expected to result in oxidative stress *in vivo* (Figure 1). First ETA is oxygenated to the corresponding sulfenic acid, ethionamide *S*-oxide (ETASO), and subsequently to a highly reactive sulfinic acid that spontaneously breaks down to 2-ethyl-4-amidopyridine (ETAA) [16,17]. Mammalian FMOs catalyze the first oxygenation with a higher K_cat_ than the second, whereas the converse is true for EtaA. Reaction mixtures containing human or mouse FMOs, NADPH, ETA and glutathione (GSH) yield less ETASO due to adduction of ETASO with GSH and redox cycling (Scheme 1) [16]. Similar reactions performed with EtaA and thiacetazone, another FMO prodrug, demonstrate GSH depletion due to formation of GSH adducts with the sulfinic acid breakdown product [5]; although *in vivo* mycothiol (MSH) would replace GSH in mycobacteria [21]. The hypothesis under test in this study was that the S-oxygenation of ETA would be impaired in mice deficient in FMO and this would impact subsequent pharmacokinetics of the parent compound and metabolites.

Hernandez *et al.* utilized a Cre/*loxP* approach to knock out the tandem *Fmo 1*, *2*, and *4* genes in mice [22]. When *Fmo1/2/4* null mice are dosed with imipramine, a drug metabolized by FMO1-dependent *N*-oxygenation, there were marked differences in drug effects and adverse health impacts of wild type versus knockout mice. The knockout mice showed pharmacological behavioral responses that did not occur in the wild type mice [23]. We are employing this *Fmo1/2/4* triple knockout model as a platform to establish the impact *in vivo * of the absence of FMO-dependent metabolism from lung. Here, we report an initial assessment of ETA metabolism and pharmacokinetics after a single oral dose. Results demonstrate that *Fmo1/2/4* knockouts exhibit reduced efficacy in the metabolism of ETA compared to wild type C57BL/6J mice. This mouse model should be suitable for studies to assess the proposed oxidative stress model in the context of the FMO2 genetic polymorphism and likely outcomes in human populations administered ETA long-term, and assessment of therapeutic efficacy in protocols employing TB-infected mice [24].

## 2. Results and Discussion

### 2.1. Linearity, Assay Precision, Accuracy, and Recovery Assessments

Standard concentrations were formulated for ETA, ETASO, ETAA, and thiobenzamide (TBZA), by addition to either mobile phase or bovine plasma; analytes were extracted following protein precipitation. The sensitivity of this assay for ETA was 0.25 µg/mL, for ETASO was 0.32 µg/mL, and for ETAA was 0.20 µg/mL. The retention times were 17.5 ± 0.5 min (ETA), 13.0 ± 0.5 min (ETASO), 11.5 ± 0.5 min (TBZA), and 6.3 ± 0.5 min (ETAA) (Figure 1A). The mean ± standard deviation (SD) of the r^2^ for ETA from 15 standard curves (eight in plasma, seven in mobile phase) was 0.98 ± 0.027; ETASO was 0.98 ± 0.033; ETAA was 0.98 ± 0.029. The percent recoveries for ETA, ETASO, ETAA, and TBZA, extracted from spiked mobile phase, ranged from 88%–102%, 88%–102%, 96%–101%, and 85%–99% respectively. The percent recoveries for ETA, ETASO, ETAA, and TBZA, extracted from spiked bovine plasma, ranged from 76%–106%, 88%–102%, 96%–102%, and 84%–105% respectively.

### 2.2. Stability

All plasma samples collected from mice were stored at −80 °C after whole-blood separation. The stability of ETA, ETASO, ETAA, and TBZA compounds in stored samples was investigated by extraction of bovine plasma aliquots spiked with 24.9, 27.3, 22.5, and 20.6 µg/mL (0.15 mM, each), respectively. After four months storage at −80 °C there was no degradation of the compounds, determined by peak area (Figure 1B). The stability of ETA suspended in Vet syrup was similarly tested. The age of the suspensions ranged from freshly made to greater than one year. The suspension solutions showed no degradation (Figure 1C,D).

### 2.3. Modeling Fmo Genotype Differences in ETA Metabolic Profiles

**Figure 2:**
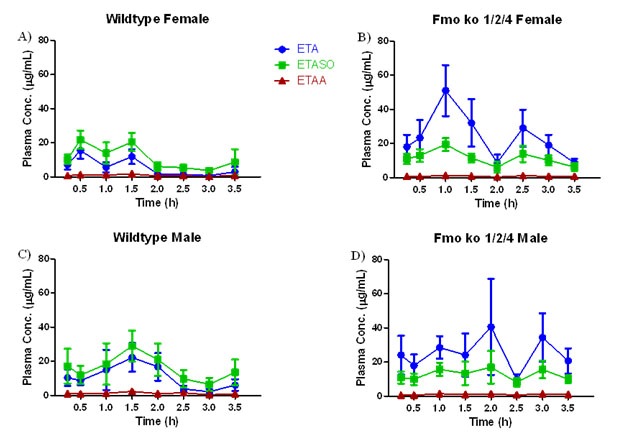
Time course for metabolism of ETA by sex and strain. Metabolic graphs depicting the means of wild type (**A**), knockout (**B**) females, and wild type (**C**), knockout (**D**) males for ETA, ETASO, and ETAA. The charts show time versus plasma concentration (µg/mL); error bars are ± SD.

A total of 144 mice were utilized to assess the pharmacokinetics of ETA and its metabolites in wild type and *Fmo*-knockout mice. Eight time points were examined, ranging from 0.25–3.5 h (Figure 2). Each mouse was gavaged with a single ETA dose of 125 mg/kg. A single 2 h time point of vehicle alone was included as a control for each eight sample time course. The time course was repeated four times for each gender and genotype (C57BL/6J (WT) or *Fmo 1/2/4* null (KO)). Vehicle control extracts displayed no interfering peaks (not shown).

**Figure 3:**
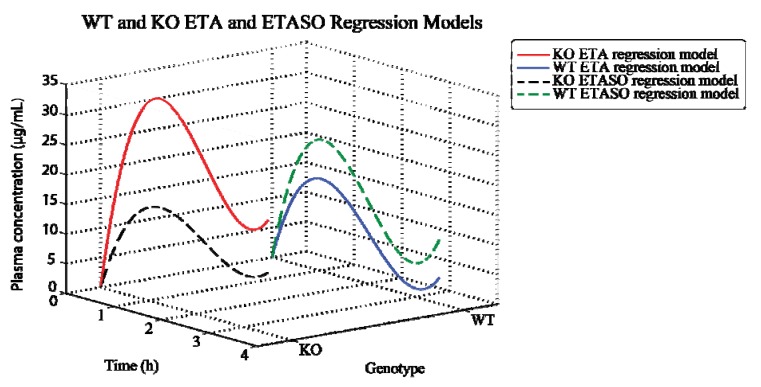
GLS regression models of ETA and ETASO plasma concentrations in KO (left) and WT (right) mice. Predicted ETA concentrations are greater than those for ETASO in KO mice, whereas predicted ETA concentrations are less than those for ETASO in WT mice, in agreement with measured ETA-ETASO plasma concentrations.

Pharmacokinetic profiles of ETA metabolism between WT and *Fmo1/2/4* knockout mice are depicted in Figure 2 and Figure 3. All mice had detectable amounts of ETAA in plasma but, levels were low in all mice and were not evaluated further. ETA concentrations were significantly greater in KO mice compared to WT (*p* < 0.001) (Figure 3).

Primary metabolite (ETASO) concentrations, as shown by GLS regression models, are greater than parent ETA concentrations in WT mice, whereas KO mice exhibited higher concentrations of ETA than ETASO (Figure 3 and Figure S2). The model of ETA and ETASO plasma concentrations (Figure 3 and Figure S1) considers ETA and ETASO observations as two separate groups; no consideration is given to the fact that each of these analytes is measured for every sample. Calculating the mean difference from individual mice generates a single value allowing modeling to consider the individual source of the paired observations. ETA-ETASO plasma concentrations in WT (−5.2 µg/mL) and KO (12.4 µg/mL) mice were significantly different (ANOVA, *p* < 0.001) in agreement with ETA and ETASO GLS regression models (Figure 3 and Figure S2), and show that the metabolic shift between ETA and ETASO levels was consistent.

#### Kinetics of ETA Metabolism in Wild Type C57BL/6J and *Fmo 1/2/4* Knockout Mice

Predicted values of pharmacokinetic measurements determined by GLS regression models are summarized by sex and genetic background in Table 1. GLS regression allows for direct comparison of genotypic differences as well as sex differences between mice. The concentration maximum (C_max_) observed in WT females for ETA was 15.5 µg/mL (predicted 15.1 µg/mL). In female KO mice the C_max_ for ETA was 51.0 µg/mL (predicted 31.0 µg/mL). The C_max_ observed in WT females for ETASO was 21.7 µg/mL (predicted 18.5 µg/mL). In female KO mice the C_max_ for ETASO was 19.4 µg/mL (predicted 16.8 µg/mL). Predicted pharmacokinetic measurements from male mice are also summarized in Table 1. The C_max_ observed in WT males for ETA was 22.3 µg/mL (predicted 15.1 µg/mL). In male KO mice the C_max_ for ETA was 40.6 µg/mL (predicted 31.0 µg/mL). The C_max_ observed in WT males for ETASO was 29.0 µg/mL (predicted 18.5 µg/mL). In male KO mice the C_max_ for ETASO was 16.9 µg/mL (predicted 16.8 µg/mL). Collectively, these data agrees with the hypothesis that impaired Fmo-dependent S-oxygenation impacts ETA pharmacokinetics.

**Table 1:** Predicted pharmacokinetic parameters of ethionamide and metabolites in mice.

Pharmacokinetic parameters		Value for mice ^a^:			
		C57BL6/J		*Fmo 1/2/4* null	
		ETA	ETASO	ETA	ETASO
Female	C_max_ (µg/mL)	15.1 ± 14.8	18.5± 20.6	31.0 ± 42.9	16.8 ± 7.9
	t_max_ (h)	1.0 ± 0.5	1.0 ± 0.5	1.0 ± 0.0	1.0 ± 1.0
Male	C_max_ (µg/mL)	15.1 ± 34.4	18.5 ± 36.0	31.0 ± 93.6	16.8 ± 28.3
	t_max_ (h)	1.0 ± 0.0	1.0 ± 0.0	1.0 ± 1.0	1.0 ± 1.0

^a^ Shown as predicted values determined by the GLS regression models with the root mean square error (RMSE).

One-third of the World population is infected with *M. tuberculosis*, with millions more being infected annually. The increased incidence of resistance to first-line antibiotics has caused health organizations to recommend second-line antibiotics as first-line intervention [2,3]. Second-line antibiotics possess less efficacy at clearing the infection, resulting in protracted treatment regimens of six months to, in the case of MDR-TB, over one year [2]. ETA intolerance, due to adverse physical side effects, results in treatment noncompliance in a significant percent of the population [2].

Toxicity from ETA occurs through *S-*oxygenation by human FMO, much like *M. tuberculosis* bioactivation of this prodrug by EtaA [5]. In humans, multiple FMOs are able to carry out this reaction. Murine FMO activation of ETA is similar to FMO activation in humans [7]. Polymorphisms in these enzymes may lead to differing concentrations of metabolites and increased incidents of adverse events occurring particularly in individuals of African/Hispanic descent. In this study, we utilized a mouse model in which FMO2 was not expressed to model the human FMO2 genetic polymorphism. 

This KO mouse, created by Hernandez *et al.* [22,23], serves as a model for individuals with the FMO2*2 genotype (all Europeans and Asians, and most individuals of African and Hispanic origin). FMO2*2 expression produces a truncated protein, (FMO2.2), unable to bind FAD, that is catalytically inactive and thus will not metabolize ETA. Mice differ from most mammals in that Fmo1 is expressed in the lung in addition to Fmo2 [16,25]. The WT C57BL/6J mouse, with a full length, enzymatically active Fmo2, models FMO2.1 expression among some individuals of African and Hispanic descent. 

Our HPLC analysis of FMO-dependent ETA oxygenation demonstrates that ETA was readily metabolized, to the sulfenic acid (ETASO) and, to a lesser degree, the sulfinic acid, as evidenced by the spontaneous breakdown to form ETAA. The ETA plasma concentrations reached the minimum inhibitory concentration (MIC) of 0.3 µg/mL and above required for *M. tuberculosis* in all mice, ensuring that 125 mg/kg is an appropriate dose to treat infection [2,3,4]. Maximum concentrations of ETA in plasma measured in mice having functioning Fmo2 were a half to a third of those in plasma of mice having no functional Fmo2. Absolute peak concentrations of ETASO in males were 50% greater than in females. The difference between genders in mice may be attributed to sex differences in expression of Fmos or cytochromes P450 (CYPs) in mice which are not found in human [26,27].

Differences in ETA/ETASO plasma ratios in KO and WT mice have significant implications in terms of therapeutic ETA administration. In WT mice, ETA is readily metabolized by FMO, with peak concentrations of ETA and the primary metabolite ETASO peaking within one hour of ETA administration. Rapid ETA metabolism, in conjunction with greater concentrations of ETASO compared to ETA, in all time points for WT mice, suggest that the largest concern in administering clinically relevant doses of ETA to healthy individuals with normal FMO2 activity would be chronic oxidative stress due to ETASO-mediated depletion of glutathione. In KO mice, however, ETA and ETASO concentrations peak two hours after ETA administration, and ETA concentrations are greater than ETASO for all measured time points. The burden of ETA metabolism in KO mice could potentially be shifted to additional FMO isoforms or other monooxygenating enzymes, such as the CYPs. Concerns in administering ETA to individuals with the FMO2*2 genotype therefore include accumulation of ETA over multiple doses, or unintentional interactions with co-administered drugs, through competitive inhibition. Further investigation into the mechanistic differences in ETA metabolism in FMO null *vs.* WT mice and how these changes relate to humans with FMO2*2 genotype is required to support or reject the validity of these hypothesized concerns.

## 3. Experimental Section

### 3.1. Chemicals

High-performance liquid chromatography (HPLC grade) reagents were purchased from Sigma-Aldrich (St. Louis, MO, USA). The vehicle for ETA gavage suspension (Vet syrup®) was from FlavoRx (Washington, DC, USA). ETASO was custom synthesized by Tjaden BioSciences (Burlington, IA, USA). ETAA was a gift from Paul R. Ortiz de Montellano (University of California, San Francisco, CA, USA) [28].

### 3.2. Animals

Male and female C57BL/6J mice were obtained from Jackson Laboratory (Sacramento, CA, USA). These mice, utilized as study controls, were delivered at 11 weeks of age to allow mice to acclimate for one week at the Laboratory Animal Resources Center (LARC) (Oregon State University, Corvallis, OR, USA) before study initiation. *Fmo1*^(^^−/^^−)^/*Fmo2*^(^^−/^^−)^/*Fmo4*^(^^−/^^−)^ [22,29] mice were backcrossed to WTC57BL/6J mice for eight generations to produce a congenic line. The line was rederived by Harlan Laboratories (Hillcrest, UK) before shipment to Oregon State University. A breeding colony was established and maintained at LARC. Mice were provided pellet diet (Purina 5053) and water *ad libitum*. Mice were kept on a 12-h light cycle under a controlled temperature between 21–22 °C. The study was approved by the local Institutional Animal Care and Use Committee.

The *Fmo* genotype was confirmed for all knockout mice on arrival at OSU (data not shown). Tested genes included *Fmo1*, *Fmo2*, *Fmo3* and *Fmo4*, using primers and PCR conditions already described [23]; the microsomal epoxide hydrolase (MEH) gene was also included as a convenient positive control; use of primers was also described elsewhere [30]. For each mouse, genes were multiplexed together. One reaction contained *Fmo2*, *Fmo4*, and *MEH* (control), whereas the other reaction included *Fmo1* and *Fmo3* (control). DirectPCR Lysis Reagent (Tail) from Viagen Biotech (Los Angeles, CA, USA) was used to lyse mouse ear punches for DNA extraction. For a 0.2-cm-diameter ear punch the addition of 49 μL DirectPCR Lysis Reagent (Tail) containing 1 μL Proteinase K (PK) solution (20 mg/mL) was sufficient. Tubes were incubated at 55 °C for 2–16 h, with periodic vortexing to assure complete lysis; the crude lysates were then incubated at 95 °C for 10 min, to denature Proteinase K. Lastly, hair was precipitated by centrifuging for 10 s. The supernatant was transferred to a fresh tube, and was centrifuged a second time for 10 s, before transferring the supernatant to a final fresh tube. A 24 µL PCR reaction contained 1 µL of lysed ear supernatant as template.

### 3.3. In Vivo Single-Dose Ethionamide Study

ETA was ground into fine powder and suspended in Vet Syrup at 12.5 µg/µL with 10 min of sonication and an initial 30 s of vortexing. Suspensions were freshly prepared each morning that mice were gavaged. Typically, nine mice (one per time point, see below) were dosed and harvested in a single morning; all work was completed between 7 am and noon. Four individuals of each sex per time point were collected constituting four replicates for a single sex of a single strain. A single dose of 125 mg ETA/kg body weight was administered orally by gavage (10 µL/g body weight). The drug was vortexed before each gavage to ensure suspension uniformity. For every 8 mice completing a time course replicate, a single mouse was gavaged with vehicle, to allow for monitoring of interfering peaks in HPLC scans. After gavaging, mice were returned to their cages for observation. At designated intervals (0.25, 0.5, 1.0, 1.5, 2.0, 2.5, 3.0, 3.5 h post-gavage for experimental samples, 2 h post-gavage for vehicle control) mice were euthanized by CO_2 _asphyxiation and exsanguinated by direct cardiac puncture. Blood was mixed with 33 IU of sodium heparin, to prevent clotting, and stored at room temperature until processed into plasma. Plasma was prepared by centrifugation of whole blood at 2,000 xg for 10 min with subsequent storage at −80 °C until analysis.

### 3.4. Sample Extraction

Plasma preparations, with volumes ranging between 90–200 µL, were spiked with 9.6 µg/mL TBZA as an internal standard. Samples were extracted at room temperature with three volumes (200 µL) of methanol with vortexing following each addition. The mixture was incubated at room temperature for 10 min, and then centrifuged for 10 min at 4 °C at 17,000 xg. Supernatant was transferred into 1.7-mL microcentrifuge tubes and evaporated to dryness under a stream of compressed nitrogen gas. Residues were resuspended in 100 µL of solvent (H_2_O-acetonitrile, 75:25). Samples were centrifuged for 3 min at room temperature at 16,300 xg, to pellet any debris. Clear supernatant was transferred into 2.5-mL Waters (Milford, PA) HPLC vials with 150-µL glass inserts, for analysis.

Stability of analytes was tested in plasma and vet syrup. ETA, ETASO, ETAA, and TBZA were spiked (0.15 mM for each compound) into bovine plasma (Innovative Research, Novi, MI, USA) and 200-µL aliquots were stored at −80 °C for four months before extraction as described above. ETA-Vet syrup suspension (12.5 µg/µL) was extracted as described above with the following modifications: the suspension was diluted 1:1 with water to reduce viscosity; the solution was diluted 150-fold to equal 16.6 µg/mL and placed into HPLC vials for subsequent analysis.

### 3.5. Quantification of Ethionamide and Metabolites

HPLC analysis was performed on a Waters 2695 separations module equipped with a 2,996 photo diode array detector and a Waters dC_18_ Atlantis^®^ (5 µm, 3.9 × 150 mm) column. The flow rate was 0.8 mL/min. The mobile phase consisted of 80% H_2_O and 20% methanol at a temperature of 35 °C, running isocratically for 20 min. ETA, ETASO, and ETAA were prepared in solvent at concentrations from 0.04 mM to 0.3 mM with TBZA at a constant concentration of 0.1 mM, in order to generate a standard curve with each HPLC run. Peak areas of analytes were determined from tracings collected at 267 nm and were used for calculations of concentrations. A broad spectra range was also collected from 210 nm and 400 nm for each standard to establish their profile, and was collected for all samples to confirm that peaks observed in the 267 nm tracing were not impure mixtures. Quantification of analytes was by comparison to the standards; concentrations of analytes were determined from ratios of analyte to internal standard. Results were analyzed with Empower software (Waters).

### 3.6. Statistical Analysis

Statistical analyses were performed using GraphPad Prism 5 [31]. A one-way analysis of variance (ANOVA) was performed to test for differences in C_max_ and t_max_ between treatment groups. Differences in mean ETA, ETASO, and ETAA plasma concentrations between male *vs.* female and knockout *vs.* wild type mice were also tested using ANOVA (α = 0.05). Generalized least squares (GLS) regression models of ETA and ETASO plasma concentrations were developed for WT and KO mice in the statistical computing program R [32]. Additive and interactive combinations of time and sex up to the third order were considered for model parameters. Parameters that did not significantly contribute to model predictions (α = 0.05) were removed from consideration, and the ETA and ETASO regression models with the lowest Akaike information criterion (AIC) score were selected for subsequent regression analysis.

## 4. Conclusions

Altogether, our results demonstrate a significant switch of ETA (parent) and ETASO (metabolite) plasma concentrations between WT and KO mice. In WT mice plasma concentrations of ETA were greater than those of ETASO, whereas in KO mice the opposite was the case, with plasma concentrations of ETASO being greater than those of the parent. This implies a genotype-dependence for the metabolism of ETA in these populations. The *Fmo 1/2/4* null mouse was employed to model the human FMO2*2 genetic polymorphism and acts as an indicator for variations of drug efficacy between ethnic groups. We have previously shown mice express Fmo1 in addition to Fmo2 at appreciable levels in lung with much smaller amounts of Fmo3 and Fmo5 [25]. Most other mammals, including humans, express only FMO2 in lung. Also, adult humans do not express FMO1 in liver but rather FMO3. FMO5 has no detectable activity toward ethionamide. Therefore, the triple knockout we employed is a good model for the human pulmonary FMO2 genetic polymorphism. Observation of active TB infection in this mouse model could provide information on the efficacy and host/pathogen relations in the bioactivation of ETA by mouse FMOs compared with bioactivation by *M. tuberculosis* monooxygenase EtaA.

## Supplementary Files

Supplementary File 1 PDF-Document (PDF, 96 KB)

## References

[1] (2011). Reported Tuberculosis in the United States, 2010.

[2] Bastian I., Colebunders R. (1999). Treatment and prevention of multidrug-resistant tuberculosis. Drugs.

[3] Thee S., Seifart H.I., Rosekranz B., Hesseling A.C., Magdorf K., Donald P.R., Schaaf H.S. (2011). Pharmacokinetics of ethionamide in children. Antimicrob. Agents Chemother..

[4] Schon T., Juréen P., Chryssanthour E., Giske C.G., Sturegård E., Kahlmeter G., Hoffner S., Angeby K.A. (2011). Wild-type distributions of seven oral second-line drugs against *Mycobacterium tuberculosis*. Int. J. Tuberc. Lung Dis..

[5] Qian L., de Montellano P.R.O. (2006). Oxidative activation of thiacetazone by the Mycobacterium tuberculosis flavin monooxygenase EtaA and human FMO1 and FMO3. Chem. Res. Toxicol..

[6] Dover L.G., Alahari A., Grataud P., Gomes J.M., Bhowruth V., Reynolds R.C., Besra G.S., Kremer L. (2007). EthA, a common activator of thiocarbamide-containing drugs acting on different mycobacterial targets. Antimicrob. Agents Chemother..

[7] Phillips I.R., Shephard E.A. (2008). Flavin-containing monooxygenases: mutations, disease and drug response. Trends Pharmacol. Sci..

[8] Furnes B., Feng J., Sommer S.S., Schlenk D. (2003). Identification of novel variants of the flavin-containing monooxygenase gene family in African Americans. Drug Metab. Dispos..

[9] Cashman J.R. (1995). Structural and catalytic properties of the mammalian flavin-containing monooxygenase. Chem. Res. Toxicol..

[10] Krueger S.K., Williams D.E. (2005). Mammalian flavin-containing monooxygenases: Structure/function, genetic polymorphisms and role in drug metabolism. Pharmacol. Ther..

[11] Dolphin C.T., Beckett D.J., Janmohamed A., Cullingford T.E., Smith R.L., Shephard E.A., Phillips I.R. (1998). The flavin-containing monooxygenase 2 gene (FMO2) of humans, but not of other primates, encodes a truncated, nonfunctional protein. J. Biol. Chem..

[12] Whestine J.R., Yueh M.-F., McCarver D.G., Williams D.E., Park C.S., Kang J.H., Cha Y.N., Dolphin C.T., Shephard E.A., Phillips I.R., Hines R.N. (2000). Ethnic differences in human flavin-containing monooxygenase 2 (FMO2) polymorphisms: detection of expressed proteins in African Americans. Toxicol. Appl. Pharmacol..

[13] Krueger S.K., Henderson M.C., Siddens L.K., VanDyke J.E., Benninghoff A.D., Karplus P.A., Furnes B., Schlenk D., Williams D.E. (2009). Characterization of sulfoxygenation and structural implications of human flavin-containing monooxygenase isoform 2 (FMO2.1) variants S195L and N413K. Drug Metab. Dispos..

[14] Krueger S.K., Siddens L.K., Martin S.R., Yu Z., Pereira C.B., Cabacungan E.T., Hines R.N., Ardlie K.G., Raucy K.L., Williams D.E. (2004). Differences in FMO2*1 allelic frequency between Hispanics of Puerto Rican and Mexican descent. Drug Metab. Dispos..

[15] Veeramah K.R., Thomas M.G., Weale M.E., Tarekegn A., Bekele E., Mendell N.R., Shephard E.A., Bradman N., Phillips I.R. (2008). The potentially deleterious functional variant flavin-containing monooxygenase 2*1 is at high frequency throughout sub-Saharan Africa. Pharmacogenet. Genomics..

[16] Henderson M.C., Siddens L.K., Morré J.T., Krueger S.K., Williams D.E. (2008). Metabolism of the anti-tuberculosis drug ethionamide by mouse and human FMO1, FMO2 and FMO3 and mouse and human lung microsomes. Toxicol. Appl. Pharmacol..

[17] Henderson M.C., Krueger S.K., Stevens J.F., Williams D.E. (2004). Human flavin-containing monooxygenase form 2 S-oxygenation: sulfenic acid formation from thioureas and oxidation of glutathione. Chem. Res. Toxicol..

[18] Nishida C.R., de Montellano P.R.O. (2011). Bioactivation of antituberculosis thioamide and thiourea prodrugs by bacterial and mammalian flavin monooxygenases. Chem. Biol. Interact..

[19] Francois A.A., Nishida C.R., de Montellano P.R., Phillips I.R., Shephard E.A. (2009). Human flavin-containing monooxygenase 2.1 catalyzes oxygenation of the antitubercular drugs thiacetazone and ethionamide. Drug Metab. Dispos..

[20] Johnston J.P., Kane P.O., Kibby M.R. (1967). The metabolism of ethionamide and its sulphoxide. J. Pharm. Pharmacol..

[21] Vilcheze C., Av-Gay Y., Attarian R., Chen B., Liu W., Alland D., Sacchettini J.C., Jacobs W.R. (2008). Mycothiol biosynthesis is essential for ethionamide susceptibility in Mycobacterium tuberculosis. Mol. Microbiol..

[22] Hernandez D., Chandan P., Janohamed A., Phillips I.R., Shephard E.A. (2006). Deletion of genes from the mouse genome using Cre/loxP technology. Methods Mol. Biol..

[23] Hernandez D., Janmohamed A., Chandan P., Omar B.A., Phillips I.R., Shephard E.A. (2009). Deletion of the mouse Fmo1 gene results in enhanced pharmacological behavioural responses to imipramine. Pharmacogenet. Genomics.

[24] Bermudez L.E., Inderlied C.B., Kolonoski P., Petrofsky M., Young L.S. (1994). Clarithromycin, dapsone, and a combination of both used to treat or prevent disseminated Mycobacterium avium infection in beige mice. Antimicrob. Agents Chemother..

[25] Siddens L.K., Henderson M.C., VanDyke J.E., Williams D.E., Krueger S.K. (2008). Characterization of mouse flavin-containing monooxygenase transcript levels in lung and liver, and activity of expressed isoforms. Biochem. Pharmacol..

[26] Falls J.G., Blake B.L., Cao Y., Levi P.E., Hodgson E. (1995). Gender differences in hepatic expression of flavin-containing monooxygenase isoforms (FMO1, FMO3, and FMO5) in mice. J. Biochem. Toxicol..

[27] Kato R., Yamazoe Y. (1992). Sex-specific cytochrome P450 as a cause of sex- and species-related differences in drug toxicity. Toxicol. Lett..

[28] Vannelli T.A., Dykman A., de Montellano P.R.O. (2002). The antituberculosis drug ethionamide is activated by a flavoprotein monooxygenase. J. Biol. Chem..

[29] Hernandez D., Melidoni A.N., Phillips I.R., Shephard E.A., Phillips I.R., Shephard E.A. (2006). Methods in Molecular Biology: Cytochrome P450 Protocols.

[30] Hayhurst G.P., Lee Y.H., Lambert G., Ward J.M., Gonzalez F.J. (2001). Hepatocyte nuclear *f*actor 4alpha (nuclear receptor 2A1) is essential for maintenance of hepatic gene expression and lipid homeostasis. Mol. Cell Biol..

[31] (2011). GraphPad Prism, version 5; Data analysis and mapping software.

[32] (2011). Statistical Computing Program R, version 2.13.1; A free software environment for statistical computing and graphics; R Core Development Team.

