# Supplementary material for: Metabolism and Pharmacokinetics of the Anti-Tuberculosis Drug Ethionamide in a Flavin-Containing Monooxygenase Null Mouse
Source: Pharmaceuticals (Basel). 2012 Oct 25;5(11):1147–59. doi: 10.3390/ph5111147 (PMC3621790; doi:10.3390/ph5111147)

Supplementary Materials

Figure S1. GLS regression models of ETA (A) and ETASO (B) plasma concentrations in WT (right) and KO (left) mice. Genotype-related differences are significant for ETA but not ETASO (α = 0.05). Individual measurements of ETA and ETASO are plotted along with their corresponding regression models to aid in model evaluation and to better visualize the distribution of measured ETA and ETASO throughout the data set.

A)

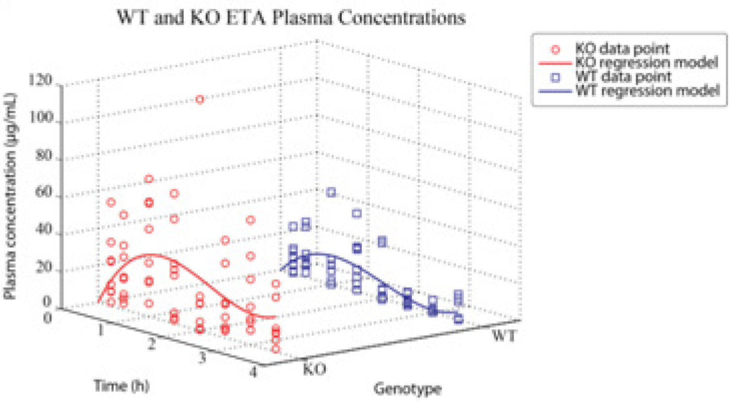

B)

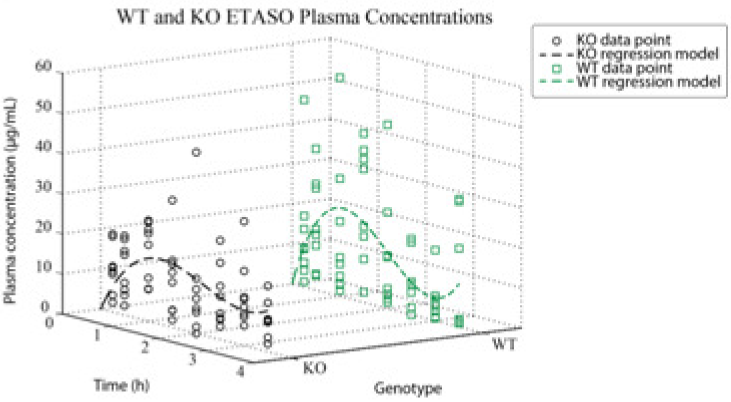

Figure S2. ETA minus ETASO plasma concentrations plotted as a function of time and genotype. Concentrations in KO mice (left) are, in general, positive (mean 12.4 μg/mL) and larger than concentrations in WT mice (right), which are, in general, negative (mean –5.2 μg/mL).

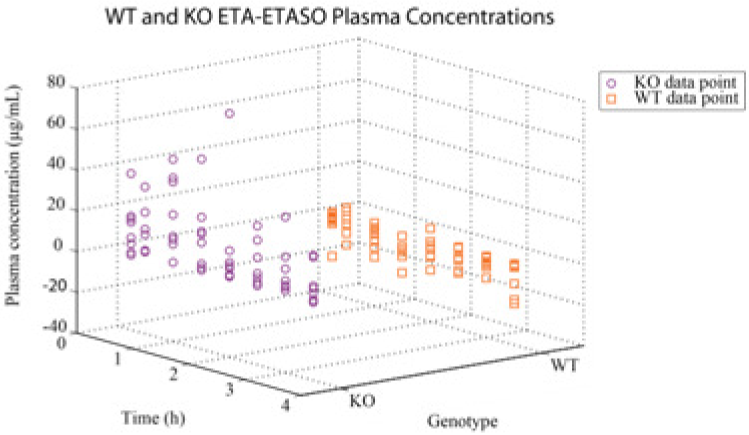

Supplement: Supplementary File 1 — PDF-Document (PDF, 96 KB) [file pharmaceuticals-05-01147-s001.pdf]
